# Early Non-Contiguous Pachymeningeal Dissemination of Isocitrate Dehydrogenase (IDH)-Wildtype Giant Cell Glioblastoma: A Rare Finding During Adjuvant Therapy

**DOI:** 10.7759/cureus.110792

**Published:** 2026-06-13

**Authors:** Elia Giacomo Galli, Fabio Spanu, Luca Emanuele, Violetta De Lucia, Bernarda Cagetti

**Affiliations:** 1 Neurosurgery, University of Genoa, Genova, ITA; 2 Neurosurgery, Ospedale Santa Corona, Pietra Ligure, ITA

**Keywords:** dural tail sign, giant cell glioblastoma, glioblastoma, idh-wildtype, ki-67 index, leptomeningeal dissemination, meningioma mimic

## Abstract

Glioblastoma multiforme (GBM) is typically characterized by a locally infiltrative presentation; secondary involvement of the dura mater and leptomeningeal disease are infrequent. A rare diagnostic finding is the isolated “dural tail sign” on contrast-enhanced MRI. While this sign is frequently considered reactive, histopathological findings in highly proliferative instances with increased extracerebral tropism confirm direct neoplastic invasion, justifying intensified surveillance in patients diagnosed with GBM. We present a case of glioblastoma, IDH-wildtype, CNS WHO grade 4, in a patient who exhibited an exceptionally aggressive clinical course. Following maximal safe resection and the standard Stupp protocol, the patient developed multifocal dural and leptomeningeal progression within three months. Radiological findings showed extensive dural thickening and a prominent dural tail sign distinct from the primary site. Histopathological and molecular analyses revealed a highly proliferative giant cell phenotype, aligning with previously documented cases of rapid neuraxis dissemination. These findings, alongside an exceptionally high Ki-67 proliferation index and IDH-wildtype status, underscore a highly aggressive biological profile predisposing the patient to atypical spread. This case highlights the potential for rapid dural dissemination in GBM, occurring within a short interval after diagnosis, surgical treatment, and chemoradiotherapy. The mechanisms underlying this spread are hypothesized to involve neuroglial propagation along pial-arachnoid margins, local seeding secondary to surgical disruption of anatomical planes, and possibly epidural spread associated with a surgical site infection. However, evidence concerning predisposing factors is currently insufficient to modify surgical techniques and perioperative care in known GBM patients. Given the dismal prognosis of leptomeningeal dissemination, early comprehensive molecular profiling and proactive neuraxis imaging are key steps to further characterize tumoral behavior.

## Introduction

Glioblastoma (GBM) is the most common and aggressive primary central nervous system malignancy in adults [1,2]. Despite standard-of-care maximal safe resection followed by adjuvant chemoradiotherapy, the prognosis remains dismal, with a median overall survival typically ranging from 12 to 21 months [1,3]. While these tumors are notorious for their diffuse, highly aggressive, and infiltrative growth into the surrounding brain parenchyma [4,5], extensive leptomeningeal or secondary dural dissemination remains an exceedingly rare phenomenon, historically documented in approximately 1% to 5% of cases [5,6].

Recent literature has identified distinct molecular and histological features within the subset of GBMs that exhibit distant leptomeningeal and dural metastases. Specifically, these tumors frequently demonstrate high tumor mutational burden (TMB), high-frequency microsatellite instability (MSI), and highly proliferative histological phenotypes [5]. Furthermore, a highly proliferative phenotype is frequently associated with rapid, widespread dissemination [5,7]. Specific histological variants, such as giant cell glioblastoma or epithelioid glioblastoma, along with key molecular signatures like TERT promoter mutations and a chromosome 7 gain/10 loss profile (+7/-10), are strongly correlated with cases of primary leptomeningeal gliomatosis (PLG) and extra-axial spread [8,9].

Biologically, these high-grade molecular alterations are thought to drive a highly aggressive, multifocal failure pattern capable of breaching the blood-brain barrier and invading extra-axial structures. Crucially, rather than representing a purely anatomical route of spread, recent paradigms suggest that dural-leptomeningeal recurrence may instead reflect the colonization of a postoperative, inflamed, and vascularly remodeled meningeal niche [10]. In this context, alterations in extracellular-matrix (ECM) integrity, localized inflammation, vascular supply, and fluid dynamics work together to modify the permissiveness of the extra-axial compartment [4,10]. PLG and secondary dural spread represent distinct clinical challenges characterized by a precipitous clinical decline; the median survival following the diagnosis of leptomeningeal or dural dissemination is exceedingly poor, ranging from just a few weeks, with some cohorts showing a median survival of only 24 days post-biopsy, to a few months [6,8]. This rapid biological progression is further complicated by a significant radiological pitfall: dural-based recurrence or spread may resemble a benign extra-axial meningioma on neuroimaging [10,11]. This mimicry is mechanically driven by the manifestation of a “dural tail sign,” a linear thickening and contrast enhancement of the meninges adjacent to the tumor that is traditionally considered highly specific for meningiomas [12,13]. Misinterpreting this radiological feature can lead to critical diagnostic delays or inappropriate preoperative interventions, such as documented cases where patients underwent preoperative endovascular embolization of the middle meningeal artery for a suspected meningioma that was ultimately confirmed as GBM postoperatively [10,14].

In this context, we report the case of a 53-year-old female who developed rapid, multifocal dural infiltration of an IDH-wildtype GBM. This case parallels the sparse literature describing rapid dural recurrence in high-grade gliomas [10,15], while highlighting the need to identify high-risk patients and to guide individualized, targeted salvage therapies [5].

## Case presentation

A 53-year-old female underwent surgical resection of a left temporo-occipital lesion in June 2025 (Figures 1A-1B; preoperative contrast-enhancing mass volume of 6.027 cm³). A single-hole craniotomy was performed at the superior margin of the left transverse sinus, and a wide, caudally hinged dural flap was reflected. Under exoscopic and microsurgical guidance, an inferior lateral occipito-temporal transulcal approach was employed to reach a solid lesion that demonstrated pathological fluorescence with 5‑ALA. Guided by fiber tracking to protect the cranially displaced inferior longitudinal fasciculus (ILF) and inferior fronto-occipital fasciculus (IFOF), the infiltrated white matter was resected until Grade 0 fluorescence was attained.

**Figure 1:**
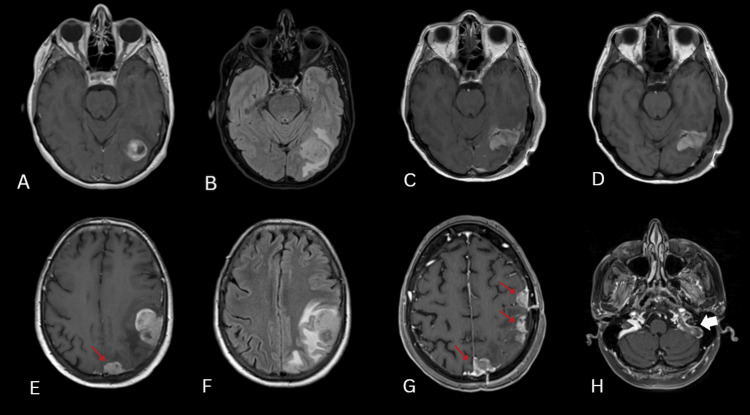
Longitudinal MRI assessment of tumor progression and postoperative changes (A, B) Initial preoperative CE-T1 and FLAIR imaging demonstrating the primary lesion. (C, D) Early postoperative CE-T1 and FLAIR imaging following the initial resection. (E, F) CE-T1 and FLAIR imaging at the time of first recurrence, highlighting non-contiguous dural spread (red arrow). (G, H) Second postoperative CE-T1 MRI showing further disease recurrence (red arrows) and associated ipsilateral sigmoid sinus thrombosis (white arrow). CE: Contrast-enhanced; FLAIR: Fluid-attenuated inversion recovery; MRI: Magnetic resonance imaging; T1: T1-weighted

Early postoperative MRI sequences confirmed a complete reduction of the contrast-enhancing tumor volume (0 cm³ residual CE; Figures 1C-1D). Although subcortical pathway preservation dictated the intentional retention of a moderate amount of residual non-contrast-enhancing FLAIR signal abnormality (Figures 1C-1D), the procedure achieved a Class 2a (Maximal CE Resection) status under the formal RANO-Resect classification system.

Given the diagnosis of glioblastoma (IDH-wildtype, WHO Grade 4, with giant cell features and an exceptionally high Ki-67 index of 70%), the patient was treated with the standard Stupp protocol (radiotherapy plus concomitant and adjuvant temozolomide). One month after resection, a wound dehiscence was observed; wound culture revealed a polymicrobial infection involving a multidrug-resistant strain of methicillin-resistant *Staphylococcus aureus* (co-resistant to fluoroquinolones, aminoglycosides, macrolides, and lincosamides) and a *Pseudomonas aeruginosa* strain susceptible to standard antipseudomonal agents. These infections induced significant localized inflammation but responded favorably to medical therapy and followed an uneventful clinical course.

Three months after resection, the patient presented with an acute onset of an expressive aphasia characterized by non-fluent speech production with preserved comprehension, episodes of confusion, and focal aware sensory seizures consisting of transient tingling paresthesias involving the right upper extremity. Neurological examination demonstrated language impairment without significant motor deficits at presentation; during subsequent progression, aphasia worsened to near-complete mutism and was accompanied by a mild right pronator drift. A CE-CT scan and subsequent CE-MRI (Figures 1E-1F) revealed multifocal dissemination of the primary lesion, with solid, heteroplastic nodular formations adhering to the dural lining. The largest lesions were located in the left posterior paramedian parietal and post-rolandic regions, suggestive of disease progression with meningeal involvement (Figure 2). In October 2025, the patient underwent surgical excision of three dural-adjacent lesions in the parietal and temporal regions. Macroscopically, the internal dural surface was adherent to yellowish neoplastic tissue; although not overtly infiltrating the meninges, this tissue was associated with extra-axial endophytic extensions at the superior parietal lobule, the parasagittal parietal region, and the temporal site of the previous surgery. Upon clinical progression, her neurological examination revealed an escalation to near-complete mutism and a new right-sided pronator drift.

**Figure 2:**
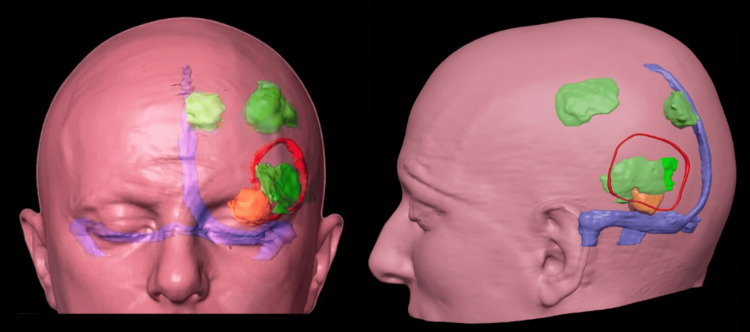
Three-dimensional (3D) reconstruction illustrating the spatial distribution of recurrent tumor and dural involvement The reconstruction demonstrates the relationship between the primary site and the pattern of spread. Orange: initial lesion; green: ultra-early contiguous and non-contiguous recurrence; red: primary craniotomy margin; blue: dural venous sinuses. 3D: Three-dimensional

The three lesions, which exhibited strong 5‑ALA fluorescence, were resected using microsurgical techniques and exoscopic visualization. Pathological fluorescence was also observed within the adjacent white matter, suggesting disease progression distant from the primary surgical site (Figure 3). Early postoperative MRI confirmed resection of the primary parietal masses but noted persistent edematous-infiltrative signal changes in the surrounding white matter. Post-contrast sequences identified a small residual or new nodular enhancement adjacent to the dural lining near the rolandic fissure and the left middle frontal gyrus, indicative of rapid multifocal dissemination (Figure 1G). Histopathological analysis of the resected dural tissue revealed fragments of meningeal-type fibrous tissue in direct continuity with GBM, consistent with the patient’s primary tumor. Microscopic analysis demonstrated marked nuclear atypia with multinucleated giant cells, focal coagulative necrosis, glomeruloid vascular proliferation, and a brisk mitotic rate reaching 8 per high-power field (8/HPF). Immunohistochemical profiling was positive for GFAP, negative for IDH1 R132H (confirming wildtype status), and showed preserved nuclear ATRX expression; the Ki-67 (MIB-1) proliferation index was exceptionally high at approximately 70% (Figure 4).

**Figure 3:**
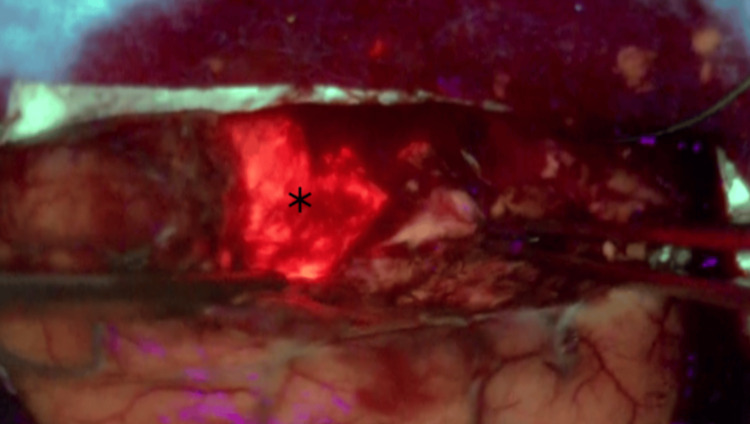
Intraoperative visualization of 5-aminolevulinic acid (5-ALA) fluorescence using the Karl Storz VITOM Eagle exoscope The image demonstrates the application of fluorescence-guided surgery to assist in the identification and resection of the tumor. The high-definition exoscopic view facilitated the visualization of fluorescence (indicated by the asterisk), aiding in the intraoperative differentiation of neoplastic tissue. 5-ALA: 5-aminolevulinic acid; VITOM: Video Telescope and Operating Monitor

**Figure 4:**
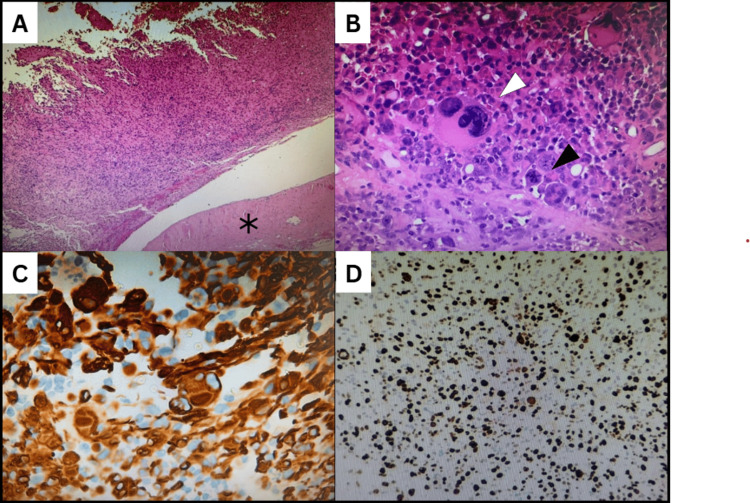
Histopathological and immunohistochemical characterization of the dural recurrence (A) Hematoxylin and eosin (H&E) staining at low magnification showing strips of meningeal-type fibrous tissue (asterisk) in continuity with hypercellular glioblastoma tissue. (B) High-magnification H&E sections demonstrating marked nuclear atypia (black arrowhead), multinucleated giant cells (white arrowhead), and focal areas of glomeruloid vascular proliferation and coagulative necrosis. (C) Glial fibrillary acidic protein (GFAP) immunohistochemistry showing strong cytoplasmic positivity, confirming the glial nature of the giant cell components. (D) Ki-67 (MIB-1) proliferation index showing a highly active, variable distribution reaching up to 70%.

Although the patient initially remained independent at home with only mild expressive aphasia, a follow-up Angio-MRI two months after the second resection revealed extensive cerebral venous sinus thrombosis involving the left sigmoid and transverse sinuses (Figure 1H). The patient was later readmitted urgently due to a sudden deterioration of expressive aphasia caused by a new intracranial hemorrhage within the surgical cavity, occurring alongside progression of the thrombosis into the jugular bulb and superior sagittal sinus. Anticoagulation was cautiously restarted following clinical stabilization; at discharge, the patient exhibited only moderate expressive aphasia. Complementary molecular analysis revealed a methylated O⁶-methylguanine-DNA methyltransferase (MGMT) promoter; further analysis for BRAF V600 mutations and NTRK fusions was requested to identify potential targeted therapy options, and salvage chemotherapy with lomustine was initiated. However, the treatment could not be completed because the patient died three months after the second surgery due to hemorrhagic complications. Table 1 shows the chronological overview of clinical evolution, multimodal diagnostic workup, and multimodal management of ultra-rapid extra-axial glioblastoma dissemination.

**Table 1:** Chronological overview of clinical evolution, multimodal diagnostic workup, and multimodal management of ultra-rapid extra-axial glioblastoma dissemination

Timeframe	Clinical Presentation & Status	Diagnostic Findings	Interventions & Outcomes
June 2025 (Baseline)	Incidental or progressive neurological symptoms leading to initial workup.	MRI: Left temporo-occipital contrast-enhancing mass (6.027 cm3).	Maximal Safe Resection (Class 2a). Early post-op MRI shows 0 cm3 residual contrast enhancement.
July 2025 (Post-Op Month 1)	Cutaneous wound dehiscence with localized inflammation.	Wound swab culture: Polymicrobial infection (MDR S. aureus & P. aeruginosa).	Successfully treated with targeted medical antibiotic therapy. Initiation of standard Stupp Protocol (RT + temozolomide).
September – October 2025 (Post-Op Months 3–4)	Acute onset of aphasia, confusion, and right upper extremity tingling paresthesias, progressing to focal deficits corresponding to mass effect from dural lesions.	September: CE-CT/CE-MRI reveal multifocal solid, heteroplastic nodular formations adhering to the dural lining (posterior paramedian parietal and post-rolandic regions).October (Intraoperative): 5-ALA positive, yellowish neoplastic tissue macroscopically adherent to the dura mater with extra-axial endophytic extensions.	Urgent diagnostic neuroimaging escalation followed by surgical excision of three dural-adjacent lesions in October. Histopathology confirms IDH-wildtype GBM with a Ki-67 index of 70%.
November 2025 (Post-Op Month 5)	Mild expressive aphasia; stable at home.	Follow-up Angio-MRI: Extensive cerebral venous sinus thrombosis involving the left sigmoid and transverse sinuses.	Initiation of salvage chemotherapy with lomustine; targeted therapies (BRAF/NTRK) requested. Cautious anticoagulation started.
December 2025 (Post-Op Month 6)	Sudden, severe clinical deterioration of expressive aphasia.	Urgent imaging: New intracranial hemorrhage within the surgical cavity and progression of thrombosis into the jugular bulb and superior sagittal sinus.	Patient readmitted urgently. Death occurs 3 months after the second surgery due to hemorrhagic complications.

## Discussion

This patient’s clinical course illustrates an unusually rapid pattern of extra-axial dissemination in IDH-wildtype glioblastoma. While glioblastomas typically recur locally at or near the resection cavity, secondary dural and leptomeningeal spread is an uncommon but devastating event, occurring in approximately 4.7-5.1% of cases following initial therapy [16,17]. Once dissemination occurs, prognosis is extremely poor, with reported median survival ranging from 39 days to 3.8 months [6,16].

The early appearance of multifocal dural-based lesions, occurring in the absence of a large local parenchymal recurrence, raises several possible mechanisms of dissemination. These include intrinsic tumor biology, cerebrospinal fluid (CSF) spread, and potential tumor cell migration facilitated by surgical manipulation. However, the relative contribution of these mechanisms cannot be determined in the present case. Any proposed explanation should therefore be regarded as hypothesis-generating rather than causal.

Several factors previously associated with leptomeningeal dissemination were present or partially present in this patient. Large cohort studies have identified younger age, larger contrast-enhancing tumor volume, proximity to the subventricular zone, surgical ventricular entry, and specific molecular profiles as risk factors for leptomeningeal metastasis [18,19]. In addition, the patient experienced a postoperative polymicrobial wound infection. Although local inflammatory changes could theoretically influence the meningeal microenvironment, there is currently insufficient evidence to establish a direct relationship between postoperative infection and dural dissemination. Consequently, any association in this case remains speculative.

During the second surgery, the recurrent lesions were found to be adherent to the dura and associated with extra-axial extensions. Histopathological examination demonstrated continuity between glioblastoma tissue and meningeal structures, confirming true dural involvement. These findings parallel previous reports describing dural-based glioblastoma recurrences that radiologically mimic meningiomas, particularly when associated with a prominent dural tail sign [10]. Recognition of this uncommon presentation is important because it may lead to diagnostic uncertainty and influence surgical planning.

The tumor exhibited giant cell features and an exceptionally high proliferative index (Ki-67 approximately 70%). Although the MGMT promoter was methylated, a factor generally associated with improved responsiveness to temozolomide, this did not prevent rapid progression. Previous studies have associated aggressive extra-axial dissemination with highly proliferative histology, IDH-wildtype status, TERT promoter alterations, and hypermutated phenotypes [5,8]. In the present case, molecular profiling confirmed IDH-wildtype status with retained ATRX expression, although broader genomic characterization was unavailable. The findings therefore support the possibility that a highly proliferative biological phenotype contributed substantially to the aggressive clinical course.

An additional feature of this case was the development of extensive cerebral venous sinus thrombosis involving the transverse and sigmoid sinuses. Although high-grade gliomas are known to promote a hypercoagulable state [9,20,21], the thrombosis likely reflected multiple interacting factors, including tumor burden, local inflammation, and mechanical effects related to dural disease. Hemorrhagic complications specifically linked to leptomeningeal dissemination remain poorly characterized [22]; in this case, the subsequent intracranial hemorrhage further complicated management and ultimately contributed to the patient’s death.

Because no validated preoperative markers reliably predict extra-axial dissemination, surgical management currently follows standard oncological principles. In selected cases with extensive dural contact or a dural tail sign, wide dural exposure, resection of visibly involved dura, watertight closure, and careful separation of surgical phases may be considered [13,23,24]. However, there is currently insufficient evidence to conclude that these measures prevent dissemination, particularly in tumors with highly aggressive biological behavior.

This report has important limitations. As a single-case observation, it cannot establish causality or definitively determine the mechanism of dissemination. It remains impossible to distinguish the relative contribution of surgical seeding, intrinsic tumor biology, CSF spread, or postoperative inflammatory changes. Furthermore, the generalizability of any preventative or management considerations derived from this case is inherently limited. Nevertheless, the case highlights the need for heightened awareness of atypical extra-axial progression patterns in highly proliferative IDH-wildtype glioblastomas; in the absence of overt dissemination, no prophylactic interventions are justified by current evidence [25].

## Conclusions

This case illustrates an unusually aggressive clinical course of IDH-wildtype glioblastoma, characterized by multifocal dural and leptomeningeal dissemination within three months of initial resection. The recurrent lesions radiologically mimicked meningioma through the presence of a prominent dural tail sign, highlighting an important diagnostic challenge.

Several mechanisms may have contributed to dissemination, including intrinsic tumor biology, cerebrospinal fluid spread, surgical factors, and local inflammatory changes. However, no definitive mechanistic conclusions can be drawn from a single case, and the relative contribution of each potential pathway remains uncertain. Likewise, the findings presented here should not be interpreted as evidence supporting specific preventative surgical modifications or perioperative management strategies.

The major limitation of this report is its inability to establish causality. Nevertheless, the case emphasizes the importance of maintaining suspicion for extracerebral dissemination in highly proliferative IDH-wildtype glioblastomas and supports the use of comprehensive molecular profiling and careful neuraxis surveillance in selected high-risk patients.
